# Deep infection in tumor endoprosthesis around the knee: a multi-institutional study by the Japanese musculoskeletal oncology group

**DOI:** 10.1186/1471-2474-14-51

**Published:** 2013-01-31

**Authors:** Takeshi Morii, Hideo Morioka, Takafumi Ueda, Nobuhito Araki, Nobuyuki Hashimoto, Akira Kawai, Kazuo Mochizuki, Shoichi Ichimura

**Affiliations:** 1 Department of Orthopaedic Surgery, Kyorin University, 6-20-2, Shinkawa, Mitaka, Tokyo 181-8611, Japan; 2 Department of Orthopaedic Surgery, Keio University, Tokyo, Japan; 3 Department of Orthopaedic Surgery, Osaka National Hospital, Osaka, Japan; 4 Department of Orthopaedic Surgery, Osaka Medical Center for Cancer and Cardiovascular Diseases, Osaka, Japan; 5 Department of Orthopaedic Surgery, Osaka University Graduate School of Medicine, Osaka, Japan; 6 Department of Musculoskeletal Oncology, National Cancer Center Hospital, Tokyo, Japan

**Keywords:** Infection, Bone tumor, Endoprosthesis, Knee

## Abstract

**Background:**

The incidence of endoprosthesis failure has been well studied, but few studies have described the clinical characteristics of deep infection in tumor prostheses. This study aimed to analyze the characteristics of deep infection in tumor endoprostheses around the knee.

**Methods:**

We analyzed clinical data of 57 patients with deep infections involving tumor endoprostheses around the knee enrolled from the Japanese Musculoskeletal Oncology Group. Profile of clinical presentation including time between surgery and infection, initial symptoms/blood tests and microbial cultures was evaluated. In addition pre-, intra-, and postoperative clinical factors influencing clinical presentation and treatment outcomes of infections were analyzed.

**Results:**

Mean interval between the initial operation and diagnosis was 13 months, and mean time required for infection control was 12 months. The most common pathogen was *Staphylococcus*. Infection control rates were significantly higher when prostheses were removed rather than salvaged. Ten-year prosthesis survival and limb salvage rates were 41.6% and 75.6%, respectively. Analysis of underlying clinical factors suggested that soft-tissue condition significantly influenced the duration of the infection control period and likelihood of limb salvage.

**Conclusions:**

Infection control is a prolonged process. Deep infection frequently results in amputation or prosthesis loss. Intensive analysis of clinical characteristics may aid infection control.

## Background

Massive endoprosthesis implantation is a conventional and convenient reconstruction modality for limb salvage operations in patients with malignant bone tumors. The implantation of these prostheses carries the risk of various complications that may require prosthesis removal or limb amputation. Prosthesis failures are classified as soft-tissue failures, aseptic loosening, structural failures, infection, and tumor progression [1]. Besides tumor progression, deep infection is the most serious of these complications and the infection rate for patients undergoing endoprosthetic implantation is between 3.6% and 44.6% [1-8]. A large amount of data has been published detailing the incidence of infection and infection-related endoprosthesis failure, including prosthesis removal and amputation. However, few data have been published describing the clinical properties of infection, including clinical symptoms at presentation, culture results, treatment modalities, and the underlying factors that might influence the characteristics of deep infections [2,9].

There are several reasonable explanations for this paucity of published data. First, the absolute number of deep infections of tumor endoprosthesis site is small in comparison to infections associated with conventional arthroplasty. For example, Hardes et al. evaluated the clinical results of 30 cases of massive prosthesis infection. Although the authors intended to examine risk factors for amputation using multivariate analysis, they concluded that there was not a large enough sample to conduct such an analysis [2]. Second, there is a wide variety of variables that might affect infections [8]. Tumor endoprostheses have broader variation in site, patient age, tumor malignancy, extent of tumor invasion, need for secure margins, patient immune condition, defect size, soft-tissue condition, and prosthesis type than conventional prostheses for total knee or hip arthroplasty. Finally, variation in the definition of deep infection used by researchers might lead to confusion in interpreting clinical results or analyzing data published in the literature [8].

In the present study, we used multiinstitutional retrospective surveillance data to analyze the conditions of tumor endoprosthesis infection around the knee, the most frequent tumor endoprosthesis infection site [10]. We focused on clinical symptoms, culture results, treatment modalities, the status of prosthesis and limb salvage, and underlying factors that might influence the characteristics of deep infections.

## Methods

We analyzed medical records from multiple treatment centers. Inclusion criteria for this study were: 1) musculoskeletal tumor around the knee that was treated at one of the 29 registered hospitals in the Japanese Musculoskeletal Oncology Group between 1995 and 2009; 2) use of an endoprosthesis in reconstruction of the limb; and 3) patients follow-up for at least 24 months or until patient death. For primary malignant tumor, wide resection was performed based on the surgical margin theory [11]. For metastatic bone tumor, margin was secured in order not to expose tumor tissue during the operation. In this study, administration of antibiotics was based on the each institutional protocol. For all except for one case, postoperative antibiotics were administrated for more than 3 days. The definition of deep infection was based on the Centers for Disease Control and Prevention guidelines [12] with the following exceptions [8]: organ-specific infection and deep incisional infection were managed together as “deep infection” and the detection period for deep infection was not limited to 12 months from the time of operation. As described by Hardes et al. [2], infections were considered controlled when there were no clinical signs of inflammation and laboratory results indicated normal C-reactive protein (CRP).

We evaluated the following variables: 1) time between surgery and infection; 2) initial symptoms/blood tests; 3) culture results; 4) infection control status; 5) number of surgical interventions for infection control; 6) modality of surgical intervention; 7) conservative therapy; 8) prosthesis survival, and 9) limb salvage. Demographic data and pre-, intra-, and postoperative factors were analyzed as independent variables that might influence characteristics of infection (Table 1). We used the definition of Hardes et al. for bad skin condition [2].

**Table 1:** Pre-, intra-, and postoperative factors of deep infection in tumor endoprostheses

**Preoperative**	
Age (years)	
Range	9–86
Mean	34.7
Median	24
Sex	
Male	32
Female	25
Comorbidity as infectious risk	
Yes	7
No	50
Tumor origin	
Primary	51
Metastatic	6
Location	
Distal femur	34
Proximal tibia	23
Grade	
Low	13
High	38
Extracompartmental tumor extension	
Yes	47
No	10
Chemotherapy	38
Radiotherapy	8
Intraoperative extraarticular resection	13
Bone resection length (cm)	
Range	10–26
Mean	15.2
Median	14
Number of antibiotics	
Single	27
Two	30
Duration of operation (min)	
Range	170–1440
Mean	376
Median	305
Intraoperative blood loss (mL)	
Range	90–10500
Mean	977.7
Median	585
Extent of partial resection of the quadriceps muscle (femur cases)	
Mean	1.8
Median	2
Muscle flap for anterior aspect of the tibia (tibia cases)	
Yes	18
No	5
Postoperative	
Bad skin condition	20
Surface infection	9
Time from surgery until infection (months)	
Range	0–85
Mean	13
Median	4
Loosening	5
Discharge/pus at infection presentation	32
Body temperature at infection presentation (°C)	
Range	35.8–40.6
Mean	38.3
Median	38.5
C-reactive protein value at infection presentation (mg/dL)	
Range	0.2–45.1
Mean	11.4
Median	9.0
Microbial culture	
*Staphylococcus aureus*	27
Methicillin-resistant *S. aureus*	10
*Staphylococcus epidermidis*	10

We used Spearman’s rank correlation coefficients, Mann–Whitney *U* tests, Fisher’s exact tests, Kaplan Meier methods and Log-rank tests to evaluate the data, as appropriate. Values of *P* less than 0.05 were considered to denote significance. Among the above-mentioned independent variables, only the factors that had significant impact on the outcomes were presented in the result. Collection of retrospective clinical data and the publication of the data were in accordance with local guidelines for research ethics and were approved by the institutional review board of the first author (H21-021, 2010/1/5).

## Results

### Demographic data

A total of 388 cases were evaluated for inclusion in this study. Of these, 57 (14.6%) were determined to have deep infections. Histological diagnosis was osteosarcoma (34 cases), metastatic bone tumor (6 cases), chondrosarcoma (4 cases), giant cell tumor of bone (3 cases), Ewing’s sarcoma (2 cases), or others (8 cases). The prostheses used were the Howmedica Modular Resection System® (36 cases), Kyocera Physio Hinge Total Knee System Type III® (13 cases), or others (8 cases).

The oncological outcomes for the infected patients included 38 continuous disease free patients, 5 patients with no evidence of disease, 6 patients who were alive with disease, 7 patients who were dead of disease, and 1 patient who died of other disease. Patients were followed for 5- to 213 months (mean, 66 months; median, 60 months). Other patient demographic data are summarized in Table 1.

### Time between surgery and infection

**Figure 1:**
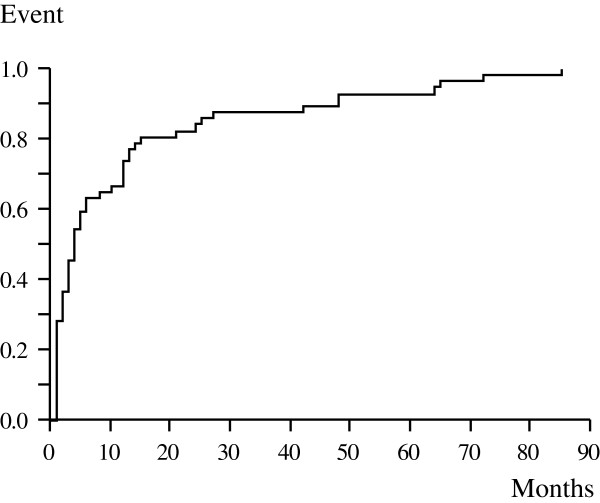
Kaplan-Meier curves showing duration from initial surgery until infection of each case.

Infection was diagnosed between 1 and 85 months (mean, 13 months; median, 4 months) after the initial surgery (Figure 1). Forty-two cases (73.7%) occurred within 12 months of surgery. The duration of the surgery–infection interval was significantly associated with tumor location and intraoperative blood loss. Infection was detected earlier in tibia cases (mean, 9 months) than in femur cases (mean, 15 months; *P* = 0.03). Time to infection was negatively associated with blood loss (*P* = 0.02).

### Initial symptoms/blood tests

Because infection was diagnosed based on clinical symptoms and blood tests, most cases had common clinical findings of deep infection such as pain, local heat, discharge/pus, local redness, and elevation of body temperature [12]. Loosening of the endoprosthesis as an initial specific symptom of infection was detected in only 5 cases (8.7%). Discharge/pus around the prosthesis was detected in 32 cases (56.1%). Body temperature at the presentation of infection ranged from 35.8°C to 40.6°C (mean, 38.3°C; median, 38.5°C). White blood cell count (per mm^3^) ranged from 300 to 18 000 (mean, 9 023; median, 8 800). In some cases, the white blood cell number was below normal range due to postoperative chemotherapy myelosuppression. CRP levels ranged from 0.2 to 45.1 mg/dL (mean, 11.4 mg/dL; median, 9.0 mg/dL). Infection occurred significantly earlier in those cases with discharge/pus than in those without (*P* = 0.03) and significantly later in those cases with loosening than in those without (*P* = 0.03). Additionally, discharge/pus was significantly more frequent among cases with extraarticular resection, than among cases without extraarticular resection (*P* = 0.03). Patients with surface infections had higher CRP values than those who did not (mean, 21.9 mg/dL vs 9.5 mg/dL; *P* = 0.009). Body temperature and white blood cell count were not significantly related to any other examined clinical or laboratory variables.

### Culture results

Pathogens were detected in 43 of the 57 cases examined (75.4%). The most frequent pathogens detected by culture included *Staphylococcus aureus* (*S. aureus*) (27 cases, 47.4%) and *Staphylococcus epidermidis (S. epidermidis)* (10 cases, 17.5%). Among the *S. aureus* cases, 10 cases were methicillin-resistant *S. aureus* (MRSA). Other pathogens included 3 cases of *Staphylococcus lugdunensis*, 3 cases of *Enterococcus* spp. (including polymicrobial infections), and 2 cases of *Pseudomonas aeruginosa*. The pathogens were not defined in 14 cases (24.6%). There were 2 cases of polymicrobial infection. Both of these cases were a combination of methicillin-sensitive *S. aureus* and *Enterococcus* spp. *S. aureus* cases were associated with extraarticular resection (*P* = 0.02), extended resection of the quadriceps muscle in the femur cases (*P* = 0.003), discharge/pus (*P* = 0.04), and elevated CRP values (*P* = 0.04). MRSA cases were associated with extraarticular resection (*P* = 0.02), prolonged operation duration (*P* = 0.004), and discharge/pus (*P* = 0.02). *S. epidermidis* cases were associated with mild inflammatory parameters (body temperature, *P =* 0.009; CRP, *P* = 0.045).

### Infection control status

**Figure 2:**
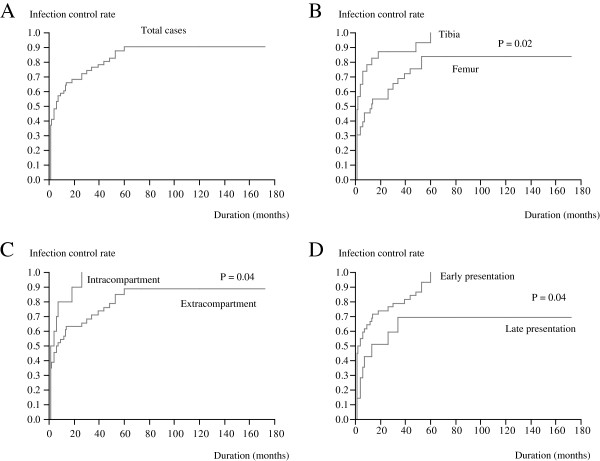
**Kaplan-Meier curves showing the duration of the interval between diagnosis of infection and the completion of infection control (A).** Factors that might influence this period include tumor location (**B**), tumor extension (**C**) and infection presentation period (**D**).

Infections were controlled in 48 cases (84.2%). The period between infection diagnosis and infection control ranged from 1 to 60 months (mean, 12 months; median, 4 months; Figure 2A). Endoprostheses in the tibia were associated with shorter infection control periods than in the femur (*P =* 0.02). Similarly, intracompartmental tumors were associated with shorter infection control periods than extracompartmental tumors (*P =* 0.04). The surgery-infection interval was negatively associated with the duration of the control period (cut off, mean presentation period, 13 months; *P =* 0.04; Figure 2B-D). In 9 patients, infection was considered as uncontrolled based on the definition of infection control, but did not require further surgical treatment—at least not until the end of the study. Of the 9 patients, 4 had fistula with acceptable general condition in terms of activities of daily life. The remaining 5 patients showed subtle (sometimes intermittent) clinical symptoms such as local heat or swelling and elevated CRP levels; these patients required continuous or intermittent administration of antibiotics despite good performance status in terms of activities of daily life.

### Number of surgical interventions for infection control

**Figure 3:**
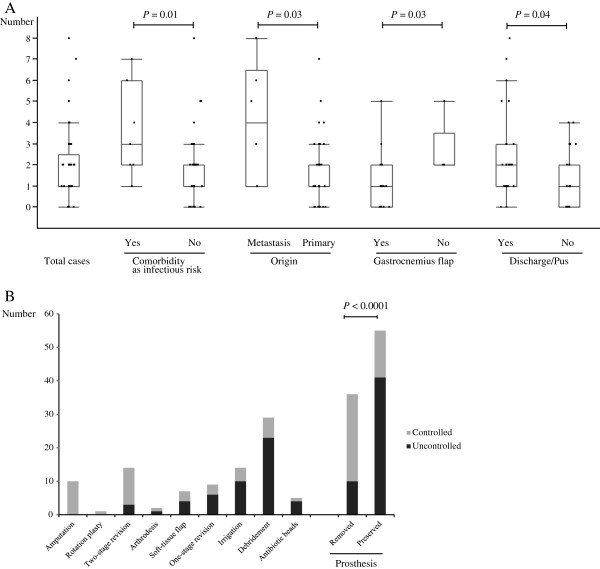
**Box-and-whisker plots showing the distribution of the number of surgical interventions required to control infection (A).** Factors significantly influencing the number of surgeries included comorbidity as infectious risk, tumor origin (primary versus metastatic), application of gastrocnemius flap (in the tibia cases), and discharge/pus at the initial presentation of infection. Success rate for each surgical modality (**B**). The modalities in this figure were arranged in order from higher to lower success rate. Modalities with removal of prostheses were significantly more successful in infection control than those that left the prostheses in place (right two rows).

Patients underwent 0 to 8 surgical interventions for infection control (mean, 1.84; median, 1; Figure 3A). Patients with comobidities that increased the risk of infection, such as diabetes mellitus (*P* = 0.01); patients of bone metastasis (*P* = 0.03); patients who lacked gastrocnemius flap coverage for a prosthesis in the tibia (*P* = 0.03); and patients with discharge/pus (*P* = 0.04), required more surgical interventions for infection control than patients without these conditions.

### Modality of surgical intervention

Overall, 91 surgeries were performed and 40 infections were controlled successfully by surgical intervention. Thus, the success rate was 44.0% for this intervention strategy. Surgical modalities and the success rate for each modality are summarized in Figure 3B. The most successful limb salvage modality was two-stage revision. Modalities with prosthesis removal (amputation, rotationplasty, two-stage revision and arthrodesis) were significantly more successful than the other treatment modalities examined (*P* < 0.0001; Figure 3B, right rows).

### Conservative therapy

Among the 48 cases with successful infection control, 8 were controlled with a conservative approach. Conservative therapy was more successful in cases with prostheses in the tibia (*P* = 0.04) and in cases without discharge/pus at the infection presentation (*P* = 0.04), than in cases with other clinical features. Of the 8 patients, 2 developed infection during postoperative chemotherapy. For these patients, infection was controlled by amelioration of myelosuppression. Among the remaining 6 patients, 5 responded to treatment with a single antibiotic agent within 2 weeks. In the other patient, 40 days were required for control with repeated changes in sensitivity, which needed re-evaluation of antibiotic effectiveness according to culture results; finally, 8 different antibiotics were used for infection control.

### Prosthesis survival

**Figure 4:**
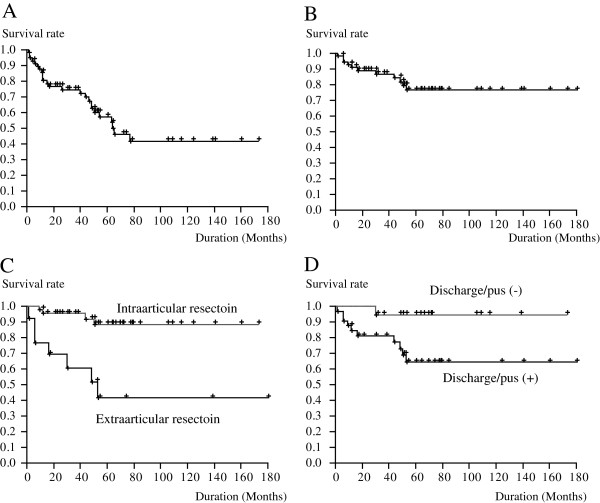
**Kaplan-Meier survival curves showing prosthesis survival (A) and limb salvage (B) in patients with deep infections after insertion of endoprostheses.** Factors that may significantly influence limb survival include extraarticular resection (**C**) and discharge/pus at the initial presentation of infection (**D**).

In 25 of the 57 cases we examined, the prostheses were removed. The 5- and 10- year survival rates were 59.3% and 39.3%, respectively (Figure 4A). Significantly more prostheses were lost in metastasis cases than in other cases (*P* = 0.009).

### Limb survival

In 11 of the 57 cases, amputation was performed. Both the 5- and 10-year survival rates were 76.9% (Figure 4B). Risk of amputation in the course of infection control was elevated by the case of metastatic bone tumor (*P* = 0.02); performance of extraarticular resection during the initial surgery (*P* = 0.0003; Figure 4C); use of 1 antibiotic, instead of 2 antibiotics, during the initial surgery (*P* = 0.04); and the presence of discharge/pus at the diagnosis of infection (*P* = 0.02; Figure 4D).

## Discussion

This study, and several preceding ones, have established several common features of deep infections of tumor endoprosthesis. These include the need for a considerably longer period of follow-up and infection monitoring for patients with endoprostheses than is required for those with conventional arthroplasty [2,13,14]; the dominant incidence of *staphylococci* as the infecting organisms [2,9,15]; the advantages of two-stage revision for infection control over other limb salvage modalities [2,9,13,14,16,17]; the difficulty of conservative therapy for deep infection [2,9]; and the need for more than 2 or more surgical interventions in order to control an infection [3,9].

Because the diagnosis of infection was based on clinical findings, we expected some homogeneity of clinical symptoms among the cases included in our study. However, we detected some variations in patient condition that might have influenced their infection condition. Early infections [2] may be associated with discharge/pus at presentation, whereas late infections [2] may be associated with prosthetic loosening. In this study, extraarticular resection, resulting in a lack of soft tissue as a protective factor against infection, was associated with pus/discharge at presentation and was predictive of poor infection control period and limb salvage prognoses (Figure 2C and 4C). This suggests that in cases with extraarticular resection, both surgeons and patients should recognize the higher risk for poor treatment outcome. In the present study, infection control rate by soft-tissue flap was 42.9%, suggesting that soft tissue coverage without prosthesis removal is not sufficient for infection control. Combination of soft tissue coverage, especially microvascular free flap coverage, and two-stage revision has been reported to yield favorable results, highlighting the importance of both soft tissue and prosthesis management in endoprosthesis infection [18]. We agree that such methods should be employed, especially in patients at high risk of poor infection control, such as those with extraarticular resection or discharge/pus at infection presentation.

*S. aureus* infections were associated with a lack of soft tissue (large defect of quadriceps muscle, extraarticular resection). Factors associated with the invasiveness of the initial operation (extraarticular resection and prolonged operation time) were especially associated with MRSA infection. In contrast, patients infected with coagulase-negative *staphylococci*, displayed considerably milder signs of infection (low body temperature and CRP) than those infected with other organisms. This suggests that infection symptoms might be, at least partly, regulated by the causal organisms. In the present study, however, negative cultures were considerably more frequent than in previous studies [2,9]. Pathogens were detected in 75.4% of our cases, whereas in previous studies, the positive culture rates were reported to be 89.7% to 93%. In addition, the incidence of polymicrobial infection was lower in the present study than in previous studies (3.5% vs 20.7-26%). We also observed a higher incidence of *S. aureus*- induced infection in the present study than was reported previously. In previous reports, coagulase-negative *Staphylococcus* was reported to be dominant over *S. aureus*[2,9]. The factors underlying such differences are unclear; however, they may be partially explained by differences in infection location, soft-tissue condition, antibiotic application, and definition of infection.

Several factors that may affect infection control status and numbers of surgical intervention were detected in the present study. Early amputation after the initial surgery may have been recorded as early infection control with fewer surgical interventions. Therefore, shorter control periods or fewer surgical interventions do not necessarily imply acceptable results for each patient. Nevertheless, the risk factors that emerged in the present study for failure of infection control or for more frequent surgery for infection control, such as tumor with the extracompartmental extension, comorbidities that increased the risk of infection, such as diabetes mellitus, and lack of a gastrocnemius flap in the tibia, still reinforce the importance of the immune system and soft-tissue condition in infection control. Unexpectedly, parameters that represent the intensity of inflammation such as body temperature or CRP levels did not predict the treatment outcome of infection. On the other hand, we emphasized the importance of other clinical key findings at presentation; discharge or pus at the infection presentation and the period of infection presentation, that might be useful for the prediction of treatment outcome such as the number of surgical intervention or treatment period for infection control.

The advantage of two-stage revision over other limb salvage modalities has been well established [2,9,13,14,16,17]. Although the success rate of one-stage revision is less than 50% [2,9], this modality can still be used for short-term infections or patients with extreme comorbidities [19,20]. Debridement without prosthesis removal is not recommended for the control of infection, as it has <5% success rate [2,9]. Because of the significant difference in the success of infection control between prosthesis removal and prosthesis preservation observed in the present study, we recommend early removal of prostheses for infection control.

If the conservative treatment can be successfully completed, patients can benefit greatly. The difficulty of conservative therapy is well accepted [2,9], and it was attempted in less than 10% of the cases we examined. Our results suggest that conservative therapy may be attempted in cases without discharge/pus, in limited circumstances.

Although limb salvage or prosthesis survival rates have been extensively reported, few data have been published regarding factors that might influence these rates. The significant of soft tissue has been well established for limb salvage. Poor skin condition, repeated surgery, radiotherapy, and lack of soft tissue are factors that may have affected limb salvage in previous reports [2,21]. In the present study, we found extraarticular resection and infection with discharge/pus to be novel risks for amputation, indicating the significance of soft tissue and early diagnosis and treatment.

When analyzing the infection of tumor endoprostheses, one should take into account the rarity of the condition, the wide variety of independent variables, and the diversity in the definition of infection. Compared with conventional arthroplasty, tumor endoprosthesis is less frequent, and the accompanying independent variables much more diverse. In addition, the definition of deep infection might be different from that in conventional arthroplasty. The guidelines of the Centers for Disease Control and Prevention [12] define deep infection or organ/space surgical site infection as infection that occurs within 1 year of surgery if an implant is in place, appears to be related to the operation, and involves deep soft tissues. However, in many previous studies, infections occurring more than 12 months after initial tumor endoprosthesis operations were interpreted as surgical site infections [2,8,9]. In order to overcome these bottlenecks, we selected a multicenter approach. In addition, we strictly defined deep infection at the beginning of the study to avoid confusion in data interpretation.

Nevertheless, the study has some limitations. First, it lacked a definite protocol for deep infection control, which resulted in wide variation in treatment modality among the institutions. The effect of each treatment modality might not have been precisely evaluated because its selection depended from the beginning on the infection status. Second, it lacked a definite protocol for administration of perioperative antibiotics. Third, the definition of infection used in the present study might have resulted in the collection of cases with a wide range of infectious conditions (i.e., from short-term infectious local findings with good general condition to fatal septic shocks). In addition, patients who died and thus did not reach the 24 months of follow-up were included; this increased the number of infection cases. However, this might introduce bias because those patients were no longer subject to the potential risk of infection. Finally, treatment decision is based on the intention of patients and surgeons; this might also introduce bias in treatment outcome and prosthesis/limb salvage status. For example, in this study, patients with metastatic bone tumor had a higher risk for amputation. This was, at least partly, due to poorer oncological prognosis, which led patients or surgeons to select less invasive treatment modalities. A future prospective study using a well-established protocol for infection control is necessary.

## Conclusions

Our study, based on a multiinstitutional survey, provides an overview of the clinical conditions associated with deep infection in tumor endoprostheses around the knee. We examined several factors that likely influence infections, including infection presentation period, clinical symptoms, culture results and status of infection control. Prosthesis removal and preservation of soft tissue are recommended for infection control.

## Abbreviations

CRP: C-reactive protein; S. aureus: *Staphylococcus aureus*; S. epidermidis: *Staphylococcus epidermidis*; MRSA: Methicillin-resistant *Staphylococcus aureus.*

## Competing interests

The authors declare that they have no competing interests.

## Authors’ contributions

TM collected the data, performed the statistical analysis and drafted the manuscript. TU collected the data and helped to draft the manuscript. HM, NA, NH, and AK collected the data. KM and SI helped to draft the manuscript. All authors have read and approved the final manuscript.

## Pre-publication history

The pre-publication history for this paper can be accessed here:

http://www.biomedcentral.com/1471-2474/14/51/prepub
